# Deeper insights into transcriptional features of cancer-associated fibroblasts: An integrated meta-analysis of single-cell and bulk RNA-sequencing data

**DOI:** 10.3389/fcell.2022.825014

**Published:** 2022-10-03

**Authors:** Anastasia N. Kazakova, Ksenia S. Anufrieva, Olga M. Ivanova, Polina V. Shnaider, Irina K. Malyants, Olga I. Aleshikova, Andrey V. Slonov, Lev A. Ashrafyan, Nataliya A. Babaeva, Artem V. Eremeev, Veronika S. Boichenko, Maria M. Lukina, Maria A. Lagarkova, Vadim M. Govorun, Victoria O. Shender, Georgij P. Arapidi

**Affiliations:** ^1^ Federal Research and Clinical Center of Physical-Chemical Medicine of Federal Medical Biological Agency, Moscow, Russia; ^2^ Moscow Institute of Physics and Technology (National Research University), Dolgoprudny, Russia; ^3^ Center for Precision Genome Editing and Genetic Technologies for Biomedicine, Federal Research and Clinical Center of Physical-Chemical Medicine of Federal Medical Biological Agency, Moscow, Russia; ^4^ Faculty of biology, Lomonosov Moscow State University, Moscow, Russia; ^5^ Faculty of Chemical-Pharmaceutical Technologies and Biomedical Drugs, Mendeleev University of Chemical Technology of Russia, Moscow, Russia; ^6^ National Medical Scientific Centre of Obstetrics, Gynecology and Perinatal Medicine named after V.I. Kulakov, Moscow, Russia; ^7^ Koltzov Institute of Developmental Biology of Russian Academy of Sciences, Moscow, Russia; ^8^ Institute of Experimental Oncology and Biomedical Technologies, Privolzhsky Research Medical University, Nizhny Novgorod, Russia; ^9^ Scientific Research Institute for Systems Biology and Medicine, Moscow, Russia; ^10^ Shemyakin–Ovchinnikov Institute of Bioorganic Chemistry of the Russian Academy of Sciences, Moscow, Russia

**Keywords:** cancer-associated fibroblasts, biomarkers, tumor microenvironment, cancer disease, single-cell transcriptomics

## Abstract

Cancer-associated fibroblasts (CAFs) have long been known as one of the most important players in tumor initiation and progression. Even so, there is an incomplete understanding of the identification of CAFs among tumor microenvironment cells as the list of CAF marker genes varies greatly in the literature, therefore it is imperative to find a better way to identify reliable markers of CAFs. To this end, we summarized a large number of single-cell RNA-sequencing data of multiple tumor types and corresponding normal tissues. As a result, for 9 different types of cancer, we identified CAF-specific gene expression signatures and found 10 protein markers that showed strongly positive staining of tumor stroma according to the analysis of IHC images from the Human Protein Atlas database. Our results give an insight into selecting the most appropriate combination of cancer-associated fibroblast markers. Furthermore, comparison of different approaches for studying differences between cancer-associated and normal fibroblasts (NFs) illustrates the superiority of transcriptome analysis of fibroblasts obtained from fresh tissue samples. Using single-cell RNA sequencing data, we identified common differences in gene expression patterns between normal and cancer-associated fibroblasts, which do not depend on the type of tumor.

## 1 Introduction

It is now increasingly accepted that cancer progression and response to chemotherapy depend not only on genetic and epigenetic variations in the tumor cells themselves but also on their microenvironment (Sahai et al., 2020). The tumor microenvironment (TME) is a heterogeneous system that consists of tumor and stromal cells, including fibroblasts, neuroendocrine, adipose, immune-inflammatory, and the blood and lymphatic vascular cells (Wang et al., 2017). Stromal cells closely interact with tumor cells, contributing to the development and progression of cancer. Cancer-associated fibroblasts (CAFs) constitute a significant part of the cells in TME (Alkasalias et al., 2018). Numerous studies show that CAFs promote tumor development by regulating several processes, including secretion of different signaling molecules, extracellular matrix remodeling, altering metabolic state, inducing chronic inflammation, and developing a pro-angiogenic and immunosuppressive microenvironment (Zhuang et al., 2019; Sahai et al., 2020). Despite the wide range of tumor-stimulating CAF functions discovered so far, there is no established definition of CAFs in the literature. Additionally, CAFs are still poorly characterized as their origin and subtypes are still unknown.

Currently, proteins such as α-smooth muscle actin (αSMA or ACTA2), platelet-derived growth factor receptor alpha (PDGFRα), platelet-derived growth factor receptor beta (PDGFRβ), fibroblast activation protein (FAP), vimentin (VIM), calvasculin (S100A4), periostin (POSTN), podoplanin (PDPN), asporin (ASPN), integrin α11β1 (ITGA11), collagen type XI alpha I chain (COL11A1), and microfibril associated protein 5 (MFAP5) are commonly used genes for identifying CAFs (Kalluri, 2016; Nurmik et al., 2020). However, these existing marker genes, which have been used to identify CAFs in various studies, are not universal for all types of cancer. Moreover, these genes are not exclusive to CAFs as they are also found to be expressed by other cells in the tumor microenvironment (Bergers and Song, 2005; Berdiel-Acer et al., 2014; Funa and Sasahara, 2014; Kalluri, 2016). The absence of specific markers for CAFs unfortunately often leads to the isolation of other cells from TME (Nurmik et al., 2020).

Another limitation in the field of CAFs’ research is the lack of accurate and detailed information about their differences relative to normal fibroblasts of healthy tissues (NFs). It has been repeatedly demonstrated that CAFs are phenotypically and functionally different from NFs due to reciprocal crosstalk between cancer cells and stromal cells (Bhowmick et al., 2004; Kalluri and Zeisberg, 2006; Erez et al., 2010). Multiple studies have highlighted the contribution of CAFs in tumor progression and metastasis (Kalluri, 2016; Sahai et al., 2020). It is noteworthy that opposed to CAFs, NFs are capable of inhibiting the proliferation and motility of adjacent cancer cells, thereby playing a tumor-suppressive role in cancer progression (Alkasalias et al., 2018). However, there are many studies that provide conflicting information regarding the differences between CAFs and NFs (Berdiel-Acer et al., 2014; Chen et al., 2014; Chen and Song, 2019; Nurmik et al., 2020). In our view, the lack of systematic information has arisen due to the considerable variability in experimental cell models and methods used to study the transcriptome of CAFs and NFs. Most often, CAFs are isolated from a heterogeneous population of tumor cells using CAF specific markers. An obvious disadvantage of this method is the difficulty in the accurate selection of CAF populations. It should be noted that in order to implement this approach, it is necessary to establish a primary culture. Several studies have shown that long-term cell cultivation leads to the loss of the primary phenotype of CAFs (Fuyuhiro et al., 2011; Wang et al., 2015). *In vitro* generation of CAFs by co-culturing NFs with cancer cell lines is an attractive alternative to the classical approach. However, due to the lack of distinguishing characteristics of CAFs and NFs, it is impossible to detect changes in fibroblast phenotype in time and accurately during co-cultivation. Another approach is to analyze the transcriptome of CAFs obtained from fresh tumor tissue samples. Initially, there is a limited amount of CAF material in the fresh tissue for isolation and sequencing. Analyzing such a small amount of material is possible only by using single-cell sequencing approaches. A significant advantage of this approach is the ability to accurately determine the population of fibroblasts among all other cells without their prior isolation.

In this study, for the first time, we provide deeper insights into transcriptional features of CAFs derived from various types of tumors. To identify candidate CAF markers, we integrated published scRNA-seq of >450,000 cells from 119 patients and 10 cancer types. Analysis of gene expression in all cells of various types of tumors enabled us to identify genes whose expression is unique to tumor fibroblasts and is absent in tumor cells and its microenvironment. Also, we investigated the transcriptomic differences between NFs and CAFs using 3 different existing approaches. We found that single-cell RNA sequencing data from fresh tumor and normal tissues is best suited for comparing the transcriptome of CAFs and NFs. By analyzing scRNA-seq data from different tumor types and normal tissues, we identified common differences in gene expression patterns between NFs and CAFs.

## 2 Materials and methods

### 2.1 Single-cell RNA-seq analysis

A collection of 10 publicly available scRNA-seq datasets were downloaded from the NCBI Gene Expression Omnibus (GEO) data repository (Table 1) (Edgar et al., 2002). If the FASTQ files were publicly available for the dataset, raw gene-barcode matrices were obtained by aligning reads to the 10X Genomics GRCh38 reference genome (refdata-Gex-GRCh38-2020-A) using CellRanger software (v. 4.0.0) (Zheng et al., 2017). Otherwise, ready-made gene-barcode matrices or text files with TPM values created by the authors were used for analysis.

**TABLE 1:** RNA-Seq data used in this study. The dataset title is used here as a dataset identifier.

I. Single-cell RNA-sequencing				
GEO data & Link	Tumor type	Number of samples		
GSE161529 Pal et al. (2021)	Breast cancer (BRCA)	31 primary tumor samples		
GSE132465 Lee et al. (2020)	Colorectal adenocarcinoma (CRC or COADREAD)	23 primary tumor samples and 10 matched normal mucosa samples		
GSE131907 Kim et al. (2020)	Lung adenocarcinoma (LUAD)	11 primary tumor samples and 11 matched normal lung samples		
GSE144236 Ji et al. (2020)	Squamous cell carcinoma (SCC)	10 primary tumor samples and 10 matched normal skin samples		
GSE138709 Min Zhang et al. (2020)	Intrahepatic cholangiocarcinoma (CHOL)	5 primary tumor samples		
GSE164690 Kürten et al. (2021)	Head and neck squamous cell carcinoma (HNSC)	15 primary tumor samples		
GSE141445 Chen et al. (2021)	Prostate adenocarcinoma (PRAD)	12 primary tumor samples		
GSE154778 Lin et al. (2020)	Pancreatic adenocarcinoma (PAAD or PDAC)	9 primary tumor samples		
GSE150321 Song et al. (2020)	Laryngeal squamous cell carcinoma (LSCC)	2 primary tumor samples		
GSE140312 Rao et al. (2020)	Gastrointestinal neuroendocrine cancer (GI-NET)	1 primary tumor sample		

**II. Bulk RNA-sequencing CAFs and NFs**				
**GEO data & Link**	**Tumor type**	**Number of samples**		**Information**
		**NFs**	**CAFs**	
GSE83314 Kaukonen et al., (2016)	Head and neck squamous cell carcinoma (HNSC)	3	3	Primary culture, number of passages unknown
GSE83834 Ishimoto et al., (2017)	Diffuse-type gastric cancer (DGC)	11	11	Primary culture, number of passages unknown
GSE83611	Breast cancer (BRCA)	6	3	Primary culture, 2 passages, normal fibroblasts at 1cm and 5 cm from tumor
GSE67945 Knuchel et al., (2015)	Colorectal cancer (CRC)	3	3	Primary culture, 4–7 passages
GSE85606 Pidsley et al. (2018), Lam et al. (2020)	Prostate cancer (PRAD)	4	4	Primary culture, 2–6 passages
GSE101665 Wiley et al. (2018), Sriram et al. (2019)	Pancreatic adenocarcinoma (PAAD or PDAC)	1 primary culture of normal pancreatic stellate cells	3	Primary culture, 5 passages

**III. Microarray gene expression data**				
**GEO data & Link**	**Description of cell lines**	**Number of samples**		**Cultivation duration**
		**Cultivation without tumor cells**	**Cultivation in the presence of tumor cells**	
GSE41678 Rajaram et al., (2013)	Four different fibroblast strains: HFFF2, HFF1, CCD1112Sk and Wi38	15	28	6 days
	Basal breast cancer cell lines: Cal51 and MDAMB231			
GSE116167 Naito et al., (2019)	Immortalized stomach fibroblasts (iNF-58 and iNF-60)	4	8	7 days
	DGC cell lines: HSC-44PE and 44As3			

Raw gene expression matrices were imported in R and processed using the Seurat R package (v. 3.4.1). A Seurat object was created for each tumor/normal tissue samples (Stuart et al., 2019). Filtering was conducted by removing cells with a small number of unique molecular identifiers (UMIs), a small or, conversely, a large number of detected genes compared to other cells in the sample, and cells with high percentages of mitochondrial genes. At least 100 detected genes and 500 UMIs were generally required for each cell, and no more than 20% mitochondrial reads were allowed per cell. However, the upper limit has been increased to 30% or lowered to 10% for a small number of samples. Next, gene expression matrices of the remaining set of high-quality cells were normalized by the “LogNormalize” method using the default scale factor of 10,000. Thereafter, the top 2000 variable genes were selected with the Seurat FindVariableFeatures and all samples of each dataset were combined using the integration method implemented in the Seurat package (Butler et al., 2018). Cell cycle scores and percentage of mitochondrial genes were used to regress out unwanted sources of variation (using Seurat Scale. Data function). Clustering was conducted with the FindClusters function using different principal components (PCs). Then the original Louvain algorithm was utilized for modularity optimization (Stuart et al., 2019). The resulting clusters were visualized with the Uniform Manifold Approximation and Projection (UMAP) and were annotated for known biological cell types using canonical marker genes (Table 2).

**TABLE 2:** Marker genes used to identify different types of cells.

Marker genes	
Fibroblasts and Myofibroblasts/Mural cells	*ACTA2, FAP, POSTN, ASPN, MFAP5, PDPN, ITGA11, PDGFRA, PDGFRB, S100A4, VIM, COL11A1*
Immune cells	*CD68, CD163, CD14, CSF1R, CD3E, CD3D, FGFBP2, CCR7, CX3CR1, CXCR1, CD19, MS4A1, IGHG1, MZB1, CD79A, KIT*
	(Markers of T-cells, B-cells, NK-cells, monocytes and macrophages, plasma cells and MAST cells.)
Endothelial cells	*PECAM1, VWF*
Epithelial cells	*EPCAM, SOX9*
	(Markers specific to certain types of cancer have also been used
	The absence of a marker gene of other cell types has been taken into account.)

To compare gene expression profiles between tumor and normal tissue fibroblasts, a pseudo-bulk approach was applied. We summarized the number of reads at the gene level of all fibroblasts in each individual patient sample. Differential expression analysis was performed with DESeq2 (v 1.28.1) (Love et al., 2014). To correct for multiple comparisons, the Benjamini-Hochberg method (FDR) was used. Changes in expression were considered significant if FDR<0.05 and |LogFC| ≥1.

### 2.2 Algorithm for detection of CAF marker genes

The first stage of selection of CAF specific marker genes was carried out using single-cell RNA-sequencing data from 10 different types of tumors (Table 1). As already described, for each type of tumor, all cells were divided into 5 types: endothelial cells, epithelial cells, immune cells, fibroblasts, myofibroblasts/mural cells. Next, for each tumor type we selected those genes that meet the following criteria:

- Gene expression is observed in more than 15% of fibroblasts or myofibroblasts/mural cells;
- Gene expression is observed in less than 5% of epithelial cells;
- Gene expression is observed in less than 5% of endothelial cells;
- Gene expression is observed in less than 1% of immune cells.

At the second stage of CAF markers selection, we used transcriptome data from 934 human tumor cell lines from the Broad-Novartis Cancer Cell Line Encyclopedia, as well as tumor transcriptomes from various patients from The Cancer Genome Atlas (TCGA) project. RNA-seq count matrices were downloaded from the Google Cloud Pilot RNA-sequencing for CCLE and TCGA open-access repository: https://osf.io/gqrz9. In each dataset, we selected only tumor cell lines or tumor types that were analyzed in the first selection step. For laryngeal squamous cell carcinoma (LSCC) and gastrointestinal neuroendocrine cancer (GINET), gene filtering was not performed at this stage due to the lack of relevant data in the two projects. In addition, since the TCGA data includes the transcriptomes of mixtures of cancer and stromal cells, we only selected samples with 90% tumor purity. Tumor purity estimates for all TCGA samples using the ABSOLUTE method were downloaded from the TCGA PanCanAtlas publications website: https://gdc.cancer.gov/about-data/publications/pancanatlas. For each of the 2 datasets, we calculated the median of gene expression in TPM (transcript per million) values among all samples of the same tumor type. Further, from the lists of genes obtained at the first stage of selection, we eliminated those genes whose median expression exceeded 2 TPM in each dataset.

For the third stage of CAF markers selection, we used publicly available single-cell RNA sequencing data from the Human Protein Atlas (HPA) project for the following normal tissues: colon, rectum, small intestine, breast, prostate, lung, liver, pancreas and skin. Accordingly, for laryngeal squamous cell carcinoma (LSCC) and head and neck squamous cell carcinoma (HNSC), gene filtering was not performed at this stage due to the lack of relevant data in the HPA project. Furthermore, for each type of tumor, we removed genes from the list that meet the following criteria:

- Gene expression is observed in more than 1% of any immune cells;
- Gene expression is observed in more than 5% of any other cells (except fibroblasts and smooth muscle cells).

As a result, we our analysis revealed a total of 414 genes that meet all the criteria above in at least one of the 10 types of tumors. For each of these genes, we visually analyzed images of stained sections of 9 different types of tumors obtained using immunohistochemistry methods (from the HPA project): colorectal cancer, breast cancer, liver cancer, lung cancer, head and neck squamous cell carcinoma, pancreatic cancer, prostate cancer, skin cancer and stomach cancer.

### 2.3 Analysis of bulk mRNA sequencing data

Published unprocessed RNA-Seq reads of NFs, CAFs and one primary culture of normal pancreatic stellate cells samples were downloaded from the NCBI Gene Expression Omnibus (GEO) data repository (Table 1) (Edgar et al., 2002). Quality filtering of the sequence reads was performed using Trimmomatic software (v. 0.38) (Bolger et al., 2014). Transcript-level abundances were quantified for each sample using a quasi-mapping approach with Salmon (v. 0.12.0) (Patro et al., 2017) on the Gencode reference transcriptome GRCh38. p12 (Harrow et al., 2012) with default parameters. Then transcript-level abundances were aggregated to the gene-level abundances using the Tximport R package (v. 1.16.1) (Soneson et al., 2015). Differential expression analysis was performed with DESeq2 (v 1.28.1) (Love et al., 2014) using the Wald test. Changes in expression were considered significant if pvalue<0.05 and |LogFC| ≥1. Importantly, in all the previous steps, each dataset was analyzed separately.

### 2.4 Analysis of microarray gene expression data

Two microarray datasets were downloaded from the NCBI Gene Expression Omnibus (GEO) data repository (Table 1) (Edgar et al., 2002). Microarray gene expression data for 4 fibroblast samples from healthy donors stimulated for 24 h without or with TNFα (accession number GSE132830) were also downloaded from the GEO repository. Raw microarray data from Affymetrix microarrays (GSE41678 and GSE132830) were processed using the oligo (v. 1.58.0) and affy (v. 1.72.0) R packages (Carvalho and Irizarry, 2010; Gautier et al., 2004) with standard parameters (quantile normalization, rma background correction). Uploaded microarray data from Agilent microarrays (GSE116167) were already normalized. Gene expression was compared between two groups of cells (normal fibroblasts cultured in the presence and absence of cancer cell lines) using the limma (v. 3.50.3) R package (Ritchie et al., 2015). To correct for multiple comparisons, the Benjamini-Hochberg method (FDR) was used. Changes in expression were considered significant if FDR < 0.05 and |LogFC| ≥ 1.

### 2.5 Transcriptome correlations

To compare the transcriptome profiles of the fibroblast samples, batch effects between datasets were eliminated using the Combat-seq function (or Combat function for microarray datasets) from sva R package (v. 3.42.0) (Leek et al., 2012; Zhang Y. et al., 2020). To compare transcriptome profiles of fibroblasts within the same dataset, the median values of the Spearman’s correlation coefficients were calculated as follows:

- median value of Spearman’s correlation coefficients between all CAF samples and all NF samples;
- the median value of the Spearman’s correlation coefficients between all CAF samples;
- the median value of the Spearman’s correlation coefficients between all NF samples.

Next, to compare every two datasets, the median values of the Spearman’s correlation coefficients were calculated as follows:

- median value of Spearman’s correlation coefficients between all CAF samples of one dataset and all NF samples of another dataset;
- the median value of the Spearman’s correlation coefficients between all CAF samples of one dataset and all CAFs samples of another dataset;
- the median value of the Spearman’s correlation coefficients between all NFs samples of one dataset and all NF samples of another dataset.

### 2.6 Functional enrichment analysis

Functional Reactome pathways enrichment analysis and gene set enrichment analysis (GSEA) were performed using the clusterProfiler R package (v. 3.16.1) (Yu et al., 2012), the ReactomePA R package (v. 1.32.0) (Yu and He, 2016) and the msigdbr (v. 7.4.1) R package (Liberzon et al., 2011) individually for each dataset. *p*-value cutoff of 0.05 were considered as statistically significant.

### 2.7 Cell culture

The human dermal fibroblast primary cultures, breast cancer cell line MDA-MB-231, prostate cancer cell line PC3, colon cancer cell line HT29 and colorectal cancer cell line Caco2 were available from a laboratory collection of cell cultures of the Federal Research and Clinical Center of Physical-Chemical Medicine of Federal Medical Biological Agency of Russia. All cell lines were incubated as adherent cultures at 37°C in a humidified atmosphere containing 5% CO_2_ and tested as mycoplasma-free. Cells at passage no older than 10 were used for analysis. The work was carried out under aseptic conditions, and cell cultures were regularly tested as mycoplasma-free.

The dermal fibroblast primary cultures, cell lines MDA-MB-231, PC3, HT29, and Caco2 were grown in DMEM supplemented with 10% FBS, 2 mM glutamine and 10 μg/ml gentamicin.

### 2.8 Fibroblasts co-culture with cancer cells

#### 2.8.1 Direct co-cultures of fibroblasts and cancer cells

The dermal fibroblasts and MDA-MB-231, PC3, HT29, or Caco2 cancer cells were cultured together on 60 mm Petri dishes at an initial ratio of 4:1 (80,000 fibroblasts and 20,000 cells of the corresponding cancer cell line) for 4 weeks without passaging. Culture medium was replaced 2 times weekly. Monocultures of fibroblasts were performed in parallel for the same duration. At the end of the co-cultivation period, fibroblasts were isolated from the co-cultures using the MACS Anti-Fibroblast MicroBeads kit (cat. #130-050-601, Miltenyi Biotec) and used immediately for total RNA isolation.

#### 2.8.2 Co-cultivation of fibroblast in the presence of conditioned medium from cancer cells

The dermal fibroblasts and MDA-MB-231, PC3, HT29, or Caco2 cancer cells were cultured separately on 60 mm Petri dishes for 4 weeks without passaging. Every 3 days the culture medium was replaced with a fresh portion of culture medium in case of cancer cells; or with a fresh culture medium diluted 50:50 with the conditioned medium from cancer cell in case of fibroblasts. Fibroblasts growing under the same conditions, but treated with conditioned media from fibroblasts itself, were used as controls.

### 2.9 RNA isolation

Total RNA was isolated from about 1 million fibroblasts or cancer cells using the RNeasy Mini Kit (cat. #74104, Qiagen) following the manufacturer’s instructions, including a DNase I treatment step (cat. #79254, Qiagen). RNA from the column was eluted with 30 µl of RNase-free water (from the kit).

Photometrical adsorption measurements at 280 nm and 260 nm confirmed purity and quantity of the purified DNase-treated RNA (NanoQuant, Tecan).

### 2.10 Reverse transcription (cDNA synthesis)

First-strand cDNA was synthesized from 4 μg of total RNA per sample in a volume of 20 µL using the SuperScript III Reverse Transcriptase (Invitrogen) and 40 pmol of random decamer primer (Evrogen) according to the manufacturer’s protocol. Experimental variation was reduced by simultaneous synthesis of cDNA for all samples.

### 2.11 Real-time quantitative RT-PCR

The oligonucleotides were used from previously published articles or designed by using Primer-BLAST (NCBI). Primer sequences are shown in Supplementary Table S1.

For each RT-qPCR reaction 4 μl of 5X qPCRmix-HS SYBR (Evrogen), 5 pmol of the respective forward and reverse primers and 1 μl of the cDNA were used. RNase-free water (Evrogen) was added to a total volume of 20 μl qRT-PCR was carried out in 0.2 ml UltraFlux i 8-Strip PCR tubes (SSI-Bio) on CFX96 Touch Real-Time PCR Detection System (Bio-Rad). All cDNA samples were tested as three or four replicates per gene and during the same run in 45 cycles (95°C for 3 min, per cycle 95°C for 10 s, 60°C for 30 s). Non-template controls and reverse transcription controls were additionally performed. A single amplification product of the expected size for each gene was verified by electrophoresis on a 1.5% agarose gel and staining with ethidium bromide.

The quantification of each PCR product was normalized to GLO1 using the 2^−ΔΔCt^ method. To select a reference gene, we considered those genes whose median expression values for NF samples and CAF samples in each dataset were at least 1 TPM. Additionally, the expression level should not differ significantly between CAFs and NFs in any dataset. Thus, GLO1 meets these requirements and has lower variance over datasets (standard-deviation log2 (TPM)) than one of the most commonly used reference genes, GAPDH (Supplementary Figure S1).

## 3 Results

### 3.1 Single-cell RNA sequencing atlas revealed transcriptional features of CAFs

**FIGURE 1:**
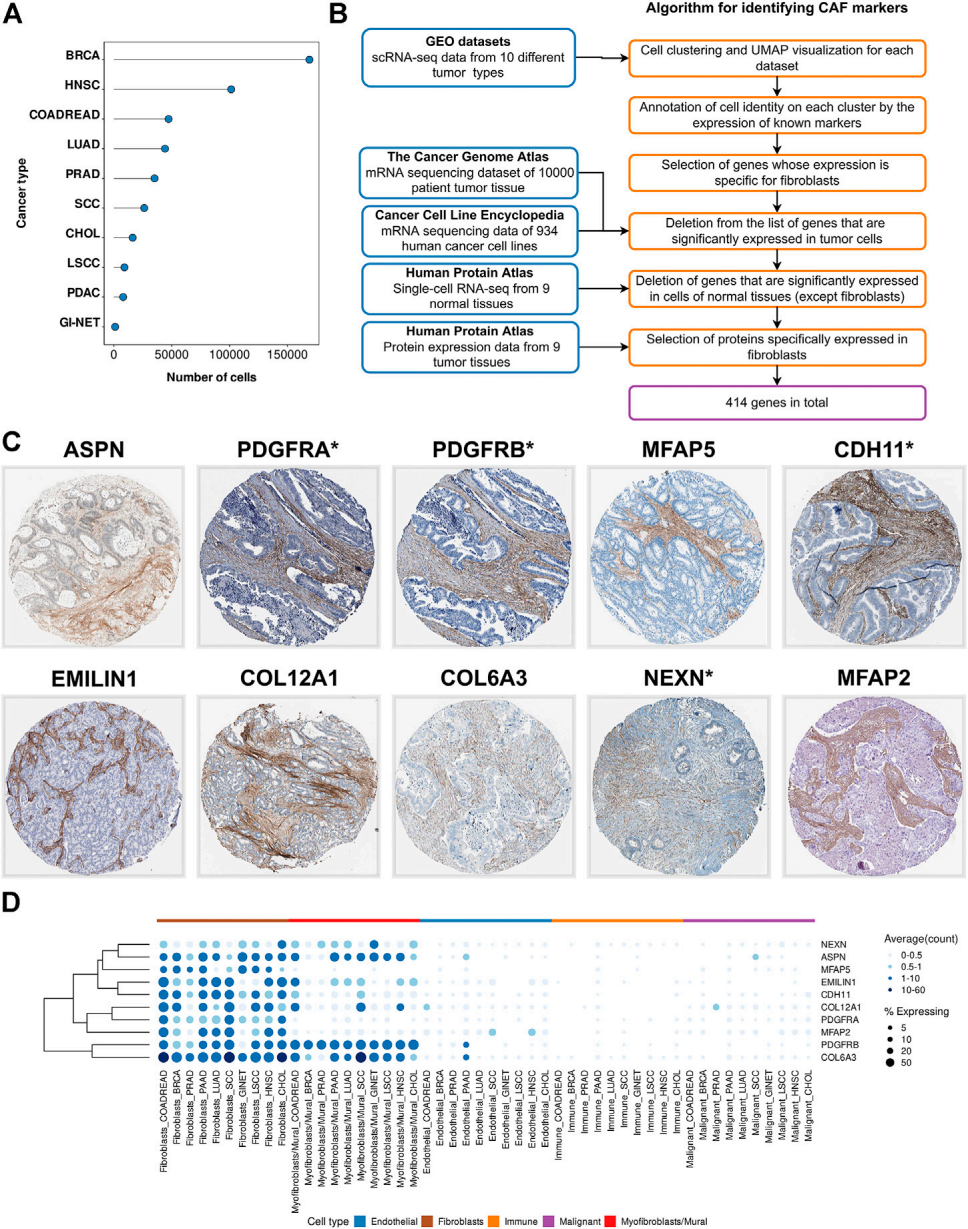
Identification of CAF markers. **(A)** The total number of analyzed cells in each of the scRNA-seq datasets. **(B)** Schematics of the study to identify CAF markers. Blue boxes indicate the datasets used in the analysis. Orange boxes indicate the algorithm used to generate significant gene list. **(C)** Immunohistochemistry (IHC) staining images showing protein expressions of ASPN, PDGFRA, PDGFRB, MFAP5, CDH11, EMILIN1, COL12A1, COL6A3, NEXN, and MFAP2 in different tumor types from the Human Protein Atlas database. **(D)** Visualization of CAF marker gene expression across cell type and tumor type. Darker color indicates higher average gene expression from the cells in which the gene was detected, and larger dot diameter indicates that the gene was detected in greater proportion of cells from the cluster. Types of tumor: BRCA—breast cancer, HNSC—head and neck squamous cell carcinoma, COADREAD—colorectal adenocarcinoma, LUAD—lung adenocarcinoma, PRAD—prostate adenocarcinoma, SCC—squamous cell carcinoma, CHOL—intrahepatic cholangiocarcinoma, LSCC—laryngeal squamous cell carcinoma, PAAD-pancreatic adenocarcinoma, GINET-gastrointestinal neuroendocrine cancer.

Compared to traditional sequencing technology, single-cell technologies have the advantage of detecting differences in cell populations, including the identification of specific cell group markers in tumor tissue (Tang et al., 2019). Therefore, we created a single cell expression atlas that includes publicly available single-cell RNA-seq (scRNA-seq) datasets of different tumor types from 119 patients (total 459,699 cells) (Figure 1A). All datasets consist of primary tumor samples collected immediately after surgery without additional *in vitro* cultivation of primary cells for the following cancer types: breast cancer (BRCA), colorectal adenocarcinoma (COADREAD), lung adenocarcinoma (LUAD), intrahepatic cholangiocarcinoma (CHOL), squamous cell carcinoma (SCC), laryngeal squamous cell carcinoma (LSCC), prostate adenocarcinoma (PRAD), head and neck squamous cell carcinoma (HNSC), pancreatic adenocarcinoma (PAAD), gastrointestinal neuroendocrine cancer (GINET) (Table 1).

To account for the transcriptional heterogeneity of fibroblasts across individual tissues, all the datasets were analyzed independently (Figure 1B). For each tumor type, we used recently developed integration methods implemented in the Seurat (Butler et al., 2018) to align the single-cell profiles from different patients. We then conducted dimensional reduction analysis to cluster the cells into groups based on the similarity of their gene expression profiles. Cell clustering revealed the presence of four major cell populations in the tumor microenvironment: epithelial cells, endothelial cells, immune cells (T-, NK-, B-, MAST-cells, monocytes and macrophages) and fibroblasts. In each dataset, CAFs were clustered by cell type rather than individual patients. Detailed data processing procedures are available in the “Materials and Methods” section.

CAF marker genes are expected to be highly expressed in fibroblasts and not expressed in other types of tumor stroma cells and cancer cells. Since the scRNA-seq data allows researchers to perform gene profiling at the individual cell level, we considered the percentage of Y cells with non-zero expression of the X gene as a measure of the specificity of the X gene for Y cells. Therefore, for each type of cancer, we selected genes that were expressed in more than 15% of fibroblasts, less than 5% of endothelial cells, less than 5% tumor cells, less than 1% immune cells (Figure 1B). As a result, we identified genes that could be proposed as markers of CAFs for 10 different types of tumors.

scRNA-seq data does not give a sense of the specificity of gene expression for CAFs relative to the incredibly heterogeneous cancer cell population in other patients with different tumor subtypes. However, to consider genes as CAF markers, we need to ensure that they are not expressed in cancer cells. Therefore, we examined the expression level of selected genes in the mRNA sequencing dataset of 934 human cancer cell lines from the Cancer Cell Line Encyclopedia (CCLE) and 10000 patient tumor tissue from The Cancer Genome Atlas (TCGA). Each of these two datasets has its own limitations. *In vitro* cultivation of cancer cell lines from CCLE likely could lead to the loss of their epithelial phenotype due to the epithelial-mesenchymal transition (Piao et al., 2017; Rice et al., 2017). To address this problem, we added mRNA sequencing dataset from TCGA project into analysis. As a result, we excluded only highly expressed in cancer cells genes from consideration according to the data from both projects (see “Materials and Methods” section for additional information). We also excluded genes with high expression in normal non-fibroblast cells according to open access Single Cell Type Atlas from the Human Protein Atlas (HPA). Altogether, analyses of scRNA-seq datasets from 10 different cancer types, RNA-seq data of the TCGA and CCLE projects, scRNA-seq data of 9 normal tissues from the open access HPA Single Cell Type Atlas, led us to a final list of 414 genes that are specifically expressed in CAFs, at least in one tumor type (Supplementary Tables S2–S12).

Transcriptomics enables examination of simultaneous expression of the entire set of protein-coding genes, which is an undoubted advantage in the identification of cell population markers. Nevertheless, to isolate CAFs from tumor tissue, it is necessary to have an idea of the abundance of proteins rather than transcripts. The standard method for visualizing proteins with a single-cell resolution is antibody-based proteomics and immunohistochemistry (IHC). IHC is a reliable method for validating cell-specific expression patterns identified by scRNA-seq data, as it reveals the expression and localization of the target-protein in the context of different cell types. Within the framework of the HPA project, the protein expression of >80% was stained in tissue sections of different tumor types. Analysis of IHC images for 320 proteins (there is no information for 94 proteins on HPA project), demonstrated that 10 proteins were showed strongly positive staining of stroma in 9 tumor types (Figure 1C). In addition, we found several proteins that are predominantly localized in the stroma of certain tumor types (Supplementary Table S2). These verifications implied that the proteins we found might be CAF-specific markers.

Among the identified 10 CAF markers, 4 proteins (ASPN, PDGFRA, PDGFRB, MFAP5) have previously been described as reliable markers. However, 6 markers had never been considered before from this perspective (CDH11, EMILIN1, COL12A1, COL6A3, NEXN, MFAP2). The most promising among these 10 proteins are PDGFRA, PDGFRB, CDH11, NEXN, as they have membrane localization and can be used for CAFs isolation by fluorescence-activated cell sorting (FACS).

### 3.2 Fibroblasts or mural cells: features of the proposed marker

As already noted, we have proposed a list of 414 marker genes, the expression of which is specific for CAFs of at least one tumor type (Table 2). It should be observed that initially, at the stage of scRNA-seq data analysis, we identified the CAF population using the expression of commonly used CAF markers (*ACTA2, FAP, POSTN, ASPN, MFAP5, PDPN, ITGA11, PDGFRA, PDGFRB, S100A4, VIM, COL11A1*). Cells that reliably expressed commonly used CAF markers formed 2 separate clusters in each tumor type (Supplementary Figure S2). We found that the expression of the *PDGFRA, FAP, COL11A1, PDPN*, and *MFAP5* genes is specific to the first cluster (Supplementary Figure S3). The remaining marker genes reveal both clusters of cells. However, *ACTA2*, the most commonly used CAF marker gene, is expressed at significantly higher levels in cells of the second cluster (Supplementary Figure S3). In addition, we found a significant expression level of *S100A4* and *VIM* in all types of cells (Supplementary Figure S3), which confirms the non-specificity of these genes for both types of fibroblasts.

By analyzing previously published scRNA-seq cancer studies, we found that researchers often identify clusters among all tumor cells that have similar expression profile to that we have identified, but the approach to the cell type annotation of these clusters differs significantly. Some researchers initially take into account the presence of pericytes and smooth muscle cells in the tumor microenvironment when annotating cell clusters. Accordingly, they identify 2 or more cell clusters: fibroblasts and smooth muscle cells/pericytes/mural cells (Hutton et al., 2021; Pal et al., 2021; Song et al., 2022). In line with these studies, we found that the second cluster of cells we identified is similar to mural cells. Furthermore, Muhl and colleagues proposed the 90-gene signature capable of discriminate fibroblasts from mural cells in scRNA-seq data analysis (Muhl et al., 2020). We found that this set of 90 gene signature was consistent with a gene signature which is distinguish the clusters we have identified. However, other researchers do not classify mural cells into a separate cluster, but annotate several different fibroblast populations, for example, inflammatory CAFs and myofibroblast-like CAFs, CAF-A and CAF-B, fibroblasts and fibroblast-like cells, etc. (Li et al., 2017; Guerrero-Juarez et al., 2022; Kim et al., 2022). In this case, we noticed that the cell clusters we identified are similar to the various fibroblast populations described in these studies, respectively. According to Muhl and colleagues, we are of the opinion that the second cluster we identified represents mural cells (Muhl et al., 2020). Given the existence of different approaches to cell annotation, we selected CAF markers based on both cell clusters. We named one cluster “Fibroblasts” and another “Myofibroblasts/Mural cells”.

To take the difference between the two cell types into consideration, we analyzed gene expression in each cluster separately. Thus, at the stage of selecting fibroblast-specific genes, we chose genes with high expression in any cell cluster. We found that the expression of 414 proposed genes is specific to either only one cluster or both (Figure 1D). For example, among 10 markers that are localized in the tumor stroma according to IHC images, 4 genes (*MFAP2, MFAP5, PDGFRA, CDH11*) are specifically represented in “Fibroblasts”, while the remaining markers are expressed in both cell types (Figure 1D). To aid in better understanding, we provide information on the expression of all 414 proposed CAF marker genes in each of the two clusters (“Fibroblasts” and “Myofibroblasts/Mural cells”), as well as in all other tumor stromal cells and cancer cells (Supplementary Tables S3–S12). Accordingly, the reader can choose markers suitable for their research, taking into account the expression of these genes in each type of tumor cells, as well as the localization of the corresponding proteins in the tissues of 9 tumor types.

### 3.3 Identification of distinctive CAF characteristics according to single-cell RNA-seq data

In the first part of our work, we identified marker genes and proteins that allow us to reliably identify the fibroblast population among all tumor cells and their microenvironment. But the ultimate goal of researchers after isolating the pure fraction of fibroblasts from tumor tissues was to identify the functional differences of these cells from fibroblasts obtained from healthy tissues. Numerous studies of NFs and CAFs can be found in the literature, revealing distinctive features of their transcriptomic profiles and hence biological functions (Fuyuhiro et al., 2011; Berdiel-Acer et al., 2014; Chen et al., 2014; Kalluri, 2016; Li et al., 2017; Nguyen et al., 2019; Druzhkova et al., 2020). However, the information is largely contradictory and inconclusive (Berdiel-Acer et al., 2014; Chen et al., 2014; Chen and Song, 2019; Nurmik et al., 2020).

**FIGURE 2:**
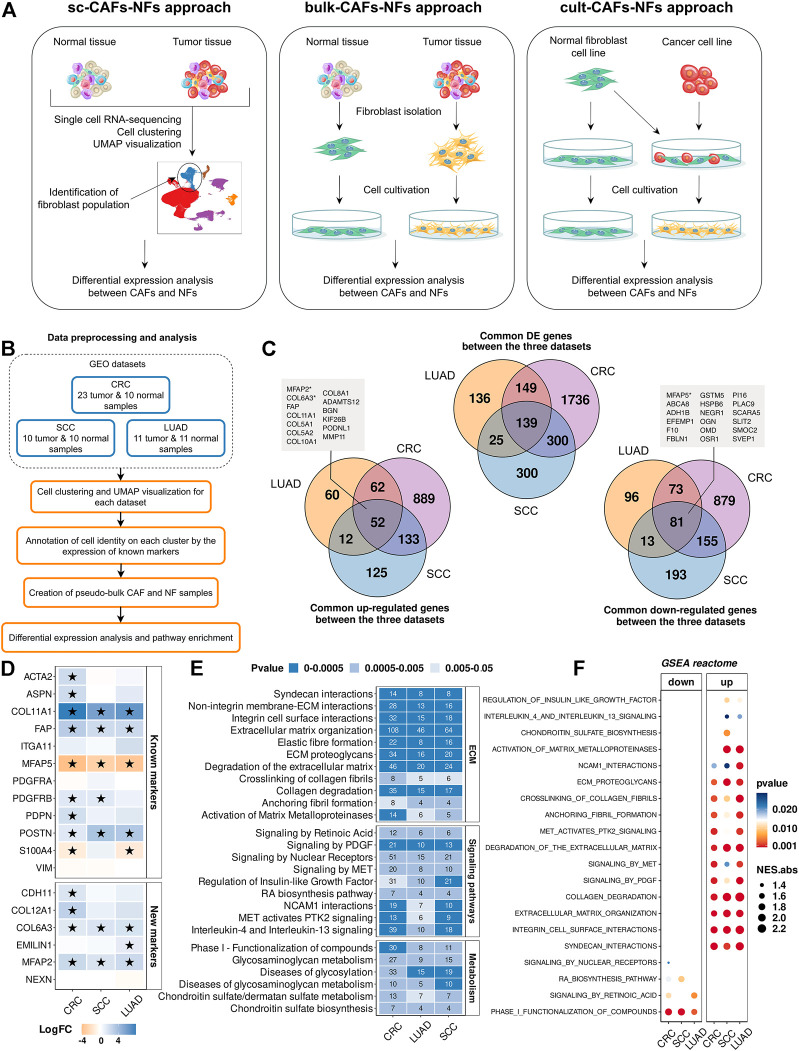
Comparison of gene expression between CAFs and NFs within the sc-CAFs-NFs approach. **(A)** Schematic diagram showing the workflow for each of the three approaches to comparing CAFs and NFs. **(B)** Workflow for comparing gene expression between CAFs and NFs by analyzing scRNA-seq data from tumors and normal tissues. **(C)** Venn diagram representing the intersection of genes differentially expressed between CAFs and NFs (upper diagram), up-regulated in CAFs (lower left diagram) and down-regulated in CAFs (lower right diagram) for colorectal cancer (purple circle), lung adenocarcinoma (yellow circle) and squamous cell carcinoma (blue circle). The boxes indicate the names of the genes that are included in the list of 414 CAF markers proposed by us. **(D)** Heatmaps showing differences in the gene expression level in CAFs relative to NFs. At the top is a heat map for common CAF marker genes, and at the bottom for our proposed CAF marker genes. Asterisks indicate significant differences in the expression (**|**logFC**|**≥1 and FDR< 0.05). Blue = increased gene expression in CAFs compared to NFs, orange = decreased gene expression in CAFs compared to NFs. **(E)** Heat map of the Reactome Pathway enrichment analysis. It represents the genes differentially expressed between CAFs and NFs. The x-axis shows the types of tumor; the y-axis shows the signaling pathways. The numbers in the boxes are the number of genes associated with the certain pathway for the given type of tumor, with a *p*-value <0.05. **(F)** Dot plots of GSEA results illustrating the enrichment of the common Reactome pathway gene sets identified by the enrichment analysis in the sc-CAFs-NFs approach. Types of tumor: CRC- colorectal cancer, LUAD—lung adenocarcinoma, SCC—squamous cell carcinoma.

To comprehensively explore the distinctive features of CAFs and NFs, we examined the transcriptomes of CAFs and NFs obtained by researchers using three different approaches (Figure 2A). Isolation of fibroblasts from tumor and corresponding normal tissues is the most commonly used method for this task. For brevity, we will call this approach bulk-CAFs-NFs, since it involves the average global gene expression analysis of all fibroblasts. Another approach we will call cult-CAFs-NFs according to the method of CAF generation (co-cultivation NFs with cancer cell lines). Both of these approaches are based on traditional sequencing methods on pooled cells cultured *in vitro*. Another approach is to analyze the transcriptome of fibroblasts obtained from fresh tumor tissue and normal tissue located at some distance from the tumor. We call this approach sc-CAFs-NF, since transcriptome analysis that does not require a large amount of cell material is possible only using single-cell sequencing approaches.

First of all, we focused on studying the transcriptome features of CAFs and NFs within the sc-CAFs-NFs approach. We selected 3 publicly available scRNA-seq datasets, which include samples of tumors and normal tissues (Figure 2B and Table 1). In total, we examined scRNA-seq expression profiles of 196787 individual cells from 75 patients spanning 3 several tumor types: colorectal cancer (CRC), lung adenocarcinoma (LUAD), and squamous cell carcinoma (SCC). All the datasets were analyzed independently as described in the first part of the work (“Single-cell RNA sequencing atlas revealed transcriptional features of CAFs” part). It should be noted that we considered only the cells of the “Fibroblasts” cluster for later analysis. To comparatively assess gene expression levels in NFs and CAFs, a pseudo-bulk approach was applied. Briefly, pseudo-bulk samples were generated by summing all reads per fibroblast cluster for each individual tissue sample.

By comparing differentially expressed genes between pseudo-bulk CAFs and NF samples, we identified 2785 protein-coding genes that were differentially expressed in at least one tumor type (|logFC|≥1 and FDR <0.05) (see “Materials and Methods” section for additional information). The vast majority of common differentially expressed genes are regulated in the same direction (co-regulated). We identified 52 genes with increased expression and 81 genes with decreased expression in all CAF samples, compared to NF samples (Figure 2C). Interestingly, among these genes, we found some of our proposed CAFs markers, including 3 markers that allow us to identify stroma cells based on IHC images - *MFAP2*, *COL6A3*, and *MFAP5* (Figure 2C). This means, changes in the expression of many of CAF markers we proposed can indicate the transition of normal fibroblasts to a cancer-associated state. Together with this, Spearman’s pairwise correlation based on the transcription profiles of fibroblast pseudo-bulk samples showed that the expression profile of the 414 marker genes proposed by us makes it possible to reliably distinguish CAF and NF samples, in contrast to the expression profile of all protein-coding genes (Supplementary Figure S4). We provided data on the gene expression changes between CAFs and NFs for each of the 414 marker genes in Supplementary Table S13. Also, among the commonly used CAF markers, we observed a significant increase in the expression level of the *COL11A1, POSTN* genes in CAFs compared to NFs (Figure 2D). In addition, the *ACTA2, ASPN, PDGFRB, PDPN, COL12A1, EMILIN1*, and *CDH11* genes were upregulated in CAFs of several tumor types (Figure 2D).

To identify biological processes responsible for the transcriptomic differences between NFs and CAFs, we analyzed the enrichment of differentially expressed genes for each dataset separately using the Reactome database. All common biological processes could be divided into three groups. The first group included the pathways involved in the rearrangement of the extracellular matrix (ECM). Specifically, we observed a significant change in the expression level of genes involved in the assembly of collagen fibrils and other multimeric structures, cell junction organization, collagen biosynthesis and activation of matrix metalloproteinases, etc., (Figure 2E). These results are in accordance with the existing opinion in the literature that CAFs play a key role in cancer progression by modifying ECM organization and stiffness (Kalluri, 2016; Sahai et al., 2020). The second group consisted of genes involved in several signaling pathways: PDGF, retinoic acid, interleukins, regulation of insulin-like growth factor, etc. (Figure 2E). The third group included genes that carry out the processes of metabolism (glycosaminoglycan metabolism, chondroitin sulfate biosynthesis, etc.) (Figure 2E). Gene Set Enrichment Analysis (GSEA) for the complete list of ranked genes showed that most of the functional changes associated with the rearrangement of the ECM and signaling pathways were up-regulated in CAFs (NES>0 and pvalue<0.05) (Figure 2F). Only 4 sets of genes were down-regulated in CAFs: signaling by nuclear receptors, signaling by retinoic acid, retinoic acid biosynthesis pathway and phase I functionalization of compounds (NES<0 and pvalue<0.05) (Figure 2F). It is worth noting once again that the sc-CAFs-NFs approach revealed the consistency of gene expression changes between CAFs and NFs for several types of tumors, which indicates similar distinctive features and functions of CAFs in various oncological diseases.

### 3.4 The bulk-CAFs-NFs approach fails to distinguish CAFs and NFs

**FIGURE 3:**
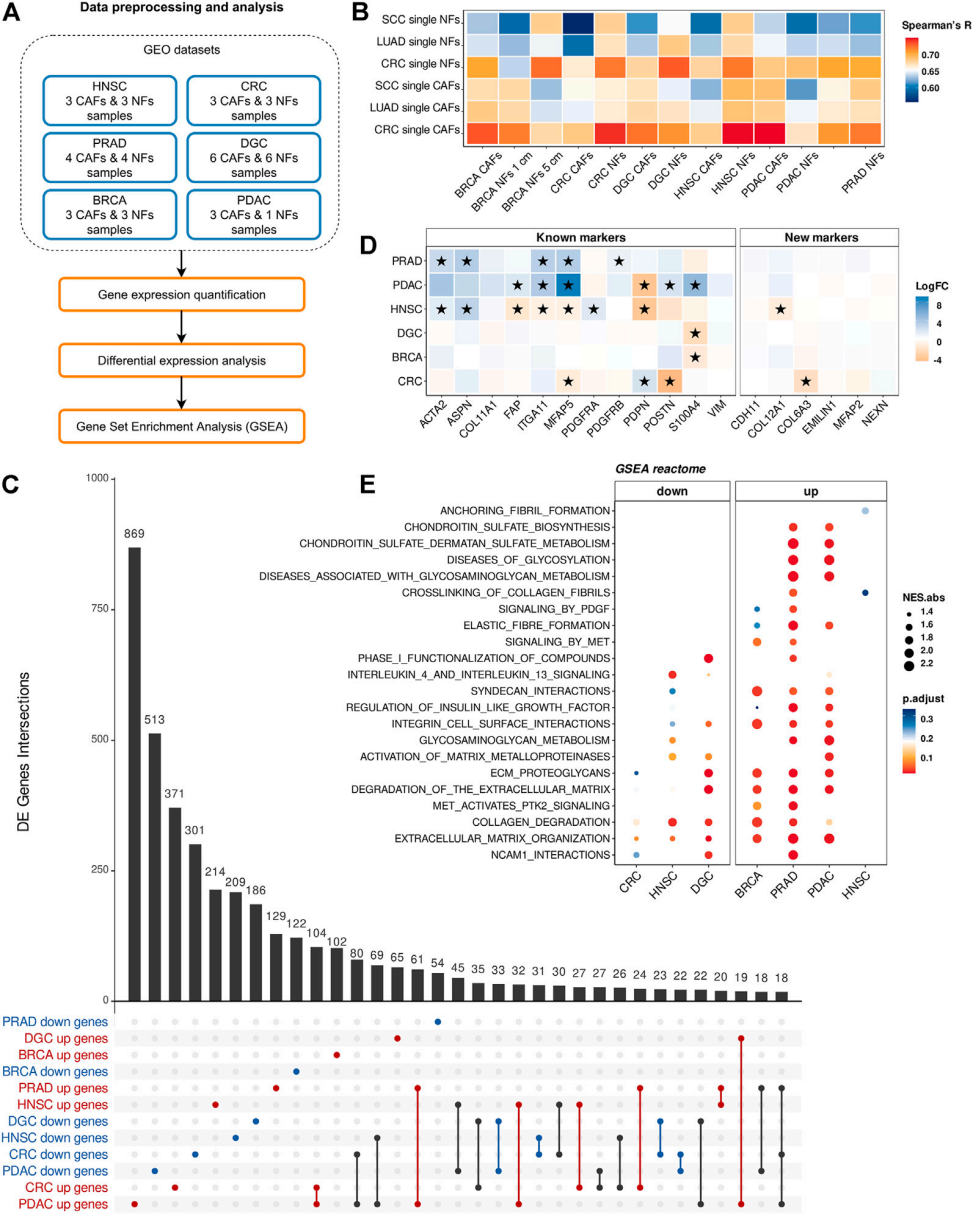
Comparison of gene expression between CAFs and NFs within the bulk-CAFs-NFs approach. **(A)** Workflow for comparing gene expression between CAFs and NFs by analyzing bulk RNA-seq data of fibroblast derived from the tumors and fibroblast derived from non-malignant regions. **(B)** The Spearman’s correlation analysis of gene expression profiles of bulk and pseudo-bulk NF and CAF samples (414 CAF marker genes). Spearman’s R values are the medians of all pairwise Spearman’s correlation coefficient values between samples. **(C)** UpSet plot indicating the number of common differentially expressed genes between CAF and NF samples for all tumor type. Gene subset intersections are highlighted in red if these genes are up-regulated in CAFs in the indicated tumor types, in blue if they are down-regulated, in black if they are down-regulated in some types of cancer and up-regulated in others. **(D)** Heatmaps showing differences in the gene expression level in CAFs relative to NFs. On the left is a heat map for common CAF marker genes, and on the right for our proposed CAF marker genes. Asterisks indicate significant differences in the expression (**|**logFC**|**≥1 and pvalue< 0.05). Blue = increased gene expression in CAFs compared to NFs, orange = decreased gene expression in CAFs compared to NFs. **(E)** Dotplots of GSEA results illustrating the enrichment of the common Reactome pathway gene sets identified by the enrichment analysis in the sc-CAFs-NFs approach. Types of tumor: HNSC—head and neck squamous cell carcinoma, DGC—diffuse-type gastric cancer, BRCA—breast cancer, CRC—colorectal cancer, PRAD—prostate cancer, PDAC—pancreatic ductal adenocarcinoma.

To quantify the differences in gene expression levels between CAFs and NFs within the bulk-CAFs-NFs approach, we collected the raw data of publicly available mRNA sequencing datasets across 6 different cancer types: head and neck squamous cell carcinoma (HNSC), diffuse gastric cancer (DGC), breast cancer (BRCA), colorectal cancer (CRC), prostate cancer (PRAD), and pancreatic ductal adenocarcinoma (PDAC) (Figure 3A). Collectively, all datasets include 22 fibroblast samples derived from the tumors, 19 fibroblast samples derived from non-malignant regions of the corresponding tissues, and one primary culture of normal pancreatic stellate cells (paired samples for PDAC CAFs) (Table 1). First of all, we examined the expression level of well-known markers of fibroblasts, endothelial cells, and epithelial cells to verify the purity of isolated fibroblasts (Berdiel-Acer et al., 2014; Kaukonen et al., 2016; Pidsley et al., 2018). All CAF and NF samples demonstrated high *ACTA2*, *VIM*, and *FAP* expression (more than 50 TPM in each sample) and low *E-cadherin*, *EPCAM* and *VE-cadherin* expression (less than 1 TPM in each sample).

In order to assess the concordance between fibroblast samples from different tissues, we performed a pairwise comparison of gene expression profiles by Spearman’s correlation. We observed a high level of correlation between the expression of all protein-coding genes for all fibroblast samples (Spearman’s R > 0.93) due to their similar phenotype. However, we did not find the expected clear separation between CAFs and NFs despite a batch correction (Supplementary Figure S5). We also expected to see a high level of Spearman’s correlation between CAF samples obtained by different approaches (sc-CAFs-NFs and cult-CAFs-NFs), and a low level of correlation between CAF and NF samples. However, comparison of transcriptome profiles of fibroblasts obtained by two different approaches showed the expected trend only for breast fibroblasts (Figure 3B).

By comparing differentially expressed genes between CAFs and NFs for each dataset, we identified 4621 protein-coding genes that were differentially expressed in at least one tumor type (**|**logFC**|**≥1 and Pvalue< 0.05). It is worth noting that not a single gene was differentially expressed between CAFs and NFs in all six datasets. Moreover, the number of common differentially expressed genes for any 3 datasets did not exceed 20 (Figure 3C). If we consider all genes that are differentially expressed in at least 2 datasets, then more than half of them are regulated in the opposite direction. In addition, we did not observe a consistent change in expression between CAFs and NFs for both the commonly used CAF markers and those proposed by us (Figure 3D).

Pathway enrichment analysis for differentially expressed genes revealed a total of 5 common biological pathways for 6 types of tumors: neuronal system, extracellular matrix organization, integrin cell surface interactions, laminin interactions, ECM proteoglycans. Since the list of statistically enriched pathways for 6 datasets showed a little overlap, we decided to investigate whether GSEA would reveal significant enrichment of those sets of genes whose expression significantly changed between CAFs and NFs in the sc-CAFs-NFs approach. For all 6 datasets, we observed an overall significant enrichment for only 2 sets of genes associated with collagen degradation and extracellular matrix organization (Figure 3E). However, in contrast to the sc-CAFs-NFs approach, this analysis revealed an inconsistency in gene expression change between CAFs and NFs for different tumor types (Figure 3E). Thus, for the bulk-CAFs-NFs approach, we did not observe consistency in changes in gene expression, as well as similarities in the biological processes that underlie the transcriptomic differences between CAFs and NFs.

### 3.5 Co-cultivation of normal fibroblasts with cancer cell lines induces proinflammatory genes expression

**FIGURE 4:**
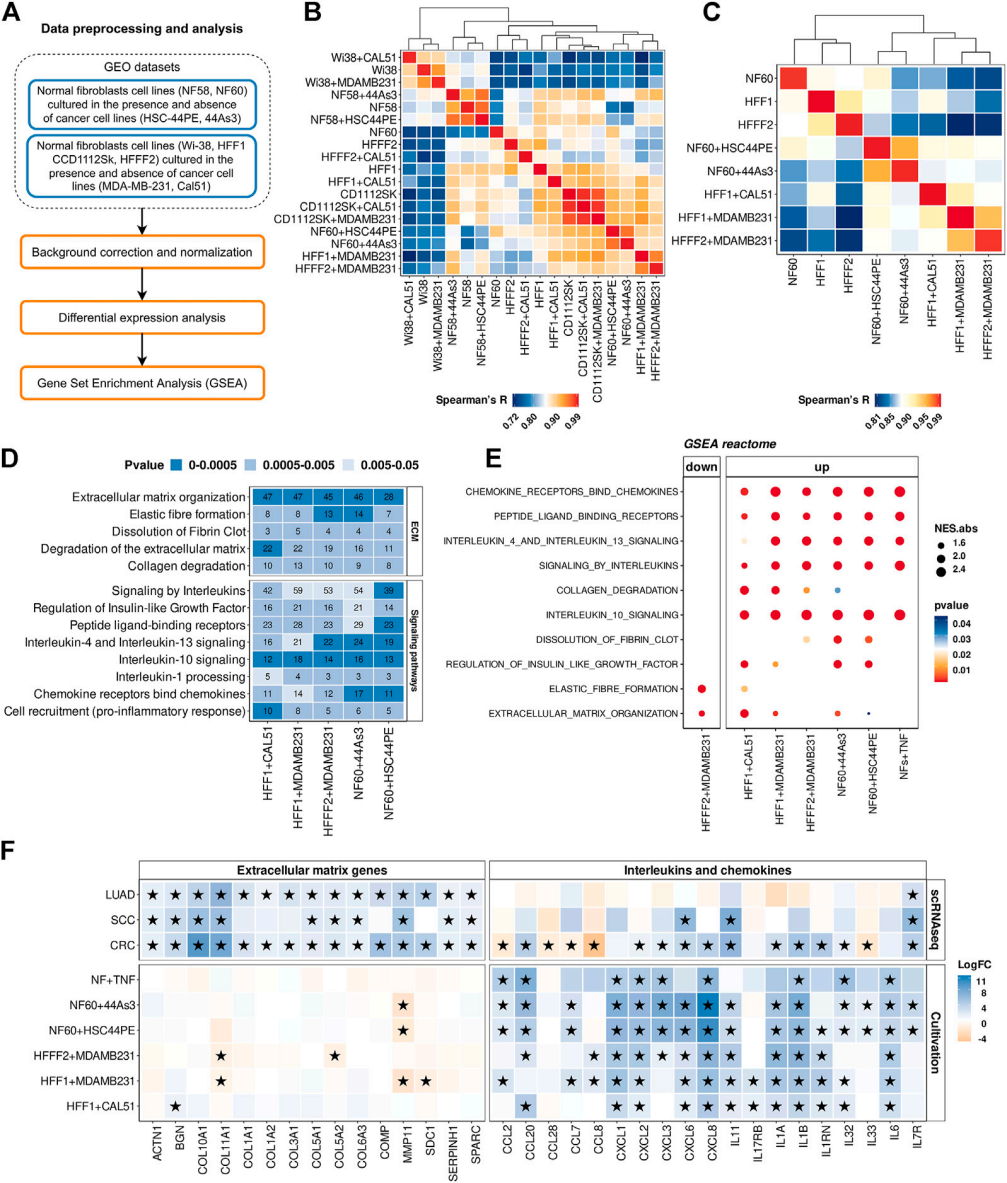
Comparison of gene expression between CAFs and NFs within the cult-CAFs-NFs approach. **(A)** Workflow for comparing gene expression between normal fibroblasts cultured in the presence and absence of cancer cell lines. **(B)** The Spearman’s correlation analysis of gene expression profiles of NF and CAF samples (414 CAF marker genes). Spearman’s R values are the medians of all pairwise Spearman’s correlation coefficient values between samples. The sample order is based on single-linked hierarchical clustering of the matrix, shown by the dendrogram. **(C)** The same Spearman correlation matrix as in point c, only for samples selected for further analysis. The sample order is based on single-linked hierarchical clustering of the matrix, shown by the dendrogram. **(D)** Heatmap of the Reactome Pathway enrichment analysis. It represents the genes differentially expressed between CAFs and NFs. The x-axis shows the types of tumor; the y-axis shows the signaling pathways. The numbers in the boxes are the number of genes associated with the certain pathway for the given type of tumor, with a *p*-value <0.05. **(E)** Dotplots of GSEA results illustrating the enrichment of the common Reactome pathway gene sets identified by the enrichment analysis in the cult-CAFs-NFs approach. **(F)** Heatmap showing differences in the gene expression level in CAFs relative to NFs in sc-CAFs-NFs and cult-CAFs-NFs approaches. On the right is a heatmap for cytokine and interleukin genes. The left heatmap is for genes involved in processes of extracellular matrix organization. Asterisks indicate significant differences in the expression (**|**logFC**|**≥1 and FDR< 0.05). Blue = increased gene expression in CAFs compared to NFs, orange = decreased gene expression in CAFs compared to NFs. Types of tumor: CRC- colorectal cancer, LUAD—lung adenocarcinoma, SCC—squamous cell carcinoma.

Within the cult-CAFs-NFs approach, we retrieved two microarray mRNA expression datasets from the GEO database (Figure 4A). In the first dataset, two immortalized stomach fibroblast cultures (NF-58 and NF-60) were co-cultured with one of two diffuse gastric cancer cell lines (HSC-44PE and 44As3). In the second study, 4 different fibroblast cultures (Wi-38, CCD1112Sk, HFFF2 and HFF1) were co-cultured with one of two basal breast cancer cell lines (MDA-MB-231 and Cal51) (Table 1). First of all, microarray expression profiles were compared using pairwise Spearman’s correlation based on the expression of all protein-coding genes as well as 414 CAF marker genes. As seen in Figure 4B and Supplementary Figure S6, samples of fibroblast cultures Wi-38, CCD1112Sk and NF-58 are grouped together regardless of culture conditions. However, some samples were separated corresponding to the culture conditions (in the presence and absence of cancer cells) (Figure 4C). It can be concluded that different fibroblast cell lines respond differently to co-cultivation with cancer cell lines within the same time period. Further analysis was performed only for the fibroblast samples indicated in Figure 4C.

**FIGURE 5:**
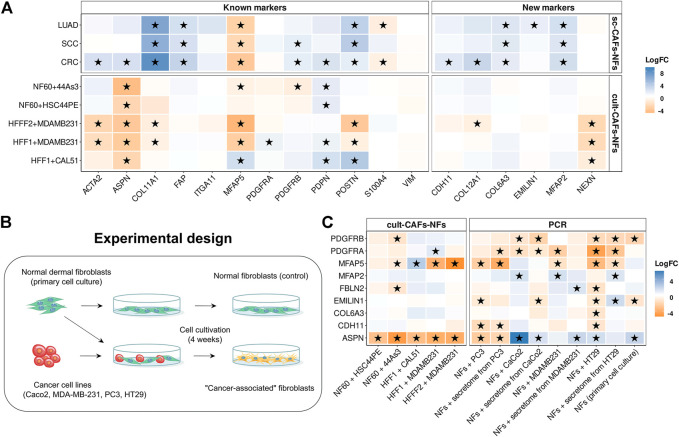
Comparison of gene expression between fibroblasts cultured with cancer cell line and mono-cultured fibroblasts. **(A)** Heatmaps showing differences in the gene expression level in CAFs relative to NFs in sc-CAFs-NFs and cult-CAFs-NFs approaches. On the left is a heat map for common CAF marker genes, and on the right for our proposed CAF marker genes. Asterisks indicate significant differences in the expression (**|**logFC**|**≥1 and FDR< 0.05). Blue = increased gene expression in CAFs compared to NFs, orange = decreased gene expression in CAFs compared to NFs. **(B)** Experimental design for comparing the expression of the proposed CAF marker genes between normal fibroblasts cultured in the presence and absence of cancer cell lines. **(C)** Heatmaps showing differences in the proposed CAF marker gene expression level in CAFs relative to NFs, based on the analysis of publicly available data (cult-CAFs-NFs approach data) and the results of our experiment (PCR). Asterisks indicate significant differences in the expression (**|**logFC**|**≥1 and FDR< 0.05 for cult-CAFs-NFs approach, **|**logFC**|**≥1 and pvalue< 0.05 for bulk-CAFs-NFs approach). Blue = increased gene expression in CAFs compared to NFs, orange = decreased gene expression in CAFs compared to NFs. Types of tumor: CRC- colorectal cancer, LUAD—lung adenocarcinoma, SCC—squamous cell carcinoma, HNSC—head and neck squamous cell carcinoma, DGC—diffuse-type gastric cancer, BRCA—breast cancer, PRAD—prostate cancer, PAAD-pancreatic adenocarcinoma.

By comparing the gene expression profile between fibroblasts cultured with cancer cell line and mono-cultured fibroblasts, we found 1446 protein-coding differentially expressed genes (|logFC|≥1 and FDR< 0.05). Enrichment of differentially expressed genes for each dataset revealed 2 groups of common biological processes (Figure 4D). As with the sc-CAFs-NFs approach, we observed a significant change in gene expression involved in different signaling pathways and rearrangement of the extracellular matrix. However, in this approach, most signaling pathways were associated with the release of interleukins and chemokines (Figure 4D). GSEA revealed similar significant up-regulation of gene sets involved in these signaling cascades (Figure 4E). Interestingly, the gene sets associated with the extracellular matrix were not significantly enriched in some culture systems (Figure 4E). Moreover, co-cultivation of the HFFF2 fibroblast cell line with the breast cancer cell line MDA-MB-231 resulted in down-regulation of genes involved in extracellular matrix organization and elastic fiber formation (Figure 4E). Consistent with this finding, many common up-regulated genes in CAFs are cytokines (Figure 4F). It is worth noting that we did not observe such a significant upregulation of the expression of interleukins and chemokines in CAFs within the sc-CAFs-NFs approach (Figure 4F). On the other hand, the expression of genes involved in extracellular matrix organization did not change in fibroblasts co-cultured with cancer cell lines versus the mono-culture (Figure 4F). Among the commonly used CAF markers, only the expression of the *PDPN* gene increased during the cultivation of normal fibroblasts with cancer cells (Figure 5A). We also found a decrease in the expression level of the *COL11A1*, *ASPN*, and *ACTA2* genes, which is inconsistent with the results of the sc-CAFs-NFs approach. Among our proposed CAF marker genes, we observed a significant change in expression under at least three cultivation conditions for the *NEXN* gene only (Figure 5A).

One of the reasons for these results may be the insufficient time of co-cultivation of fibroblasts with cancer cell lines. In the datasets we considered, the duration of cultivation did not exceed 6–7 days. To check whether the expression profile of the marker genes we proposed changes with longer cultivation, we co-cultivated normal skin fibroblasts with 4 different tumor cell lines (PC3, Caco-2, MDA-MB-231, HT-29) for 4 weeks (Figure 5B). We used both direct co-cultivation of fibroblasts with cancer cells and cultivation with secretomes from the same cancer cells. We observed the same change in gene expression during cultivation of normal fibroblasts either with the tumor cell line PC3 or with its secretome. For other cell lines, changes in the level of gene expression during direct and indirect cultivation do not always coincide (Figure 5C). It can be concluded that the presence of direct contact with tumor cells has a greater effect on the change in the fibroblast phenotype. We also noted a change in the expression level of 3 genes (*PDGFRB*, *EMILIN1* and *ASPN*) during cultivation of the primary cell line of skin fibroblasts in the absence of cancer cells for 4 weeks (Figure 5C). This suggests that the cultivation conditions themselves lead to a change in the level of expression of some genes in normal fibroblasts.

In contrast to the results obtained by other researchers, the results of our study showed a significant increase in the expression level of the *ASPN* gene in fibroblasts (|logFC|≥1 and pvalue<0.05) after cultivation with 3 different tumor cell lines (Figure 5C). However, during the cultivation of fibroblasts with the PC3 cell line, the level of *ASPN* expression decreased. We also observed an increase in *MFAP2* gene expression and a decrease in *MFAP5* gene expression in fibroblasts for some culture systems (Figure 5C). This is consistent with the results of the sc-CAFs-NFs approach. However, the expression of the *PDGFRA*, *PDGFRB*, *CDH11*, and *EMILIN1* genes was mainly reduced in fibroblasts after co-cultivation with cancer cells. In addition, during the cultivation of fibroblasts with the HT-29 cell line, the expression of most of the markers we proposed decreased (including the expression of the *COL6A3* gene, which was proposed as a distinguishing marker gene between CAFs and NFs in the sc-CAFs-NFs approach) (Figure 5C). Thus, the approach based on co-cultivation of normal fibroblasts with cancer cells does not result in complete conversion of normal fibroblasts to the cancer-associated state that is characteristic of native CAFs.

It is difficult to say whether the observed increase in the expression of interleukins and chemokines is due to the transition of normal fibroblasts to a cancer-associated phenotype or the response of cells to stress. Interestingly, we found that similar cytokine activation can be observed when normal fibroblasts are stimulated with tumor necrosis factor-α (TNFα) (Figures 4E,F). The absence of changes in the expression of ECM genes during cultivation of normal fibroblasts with cancer cells may be due to the fact that the cult-CAFs-NFs approach does not allow taking into account the influence of other cells and factors of the tumor microenvironment on fibroblasts. Altogether, the results of the analysis of the cult-CAFs-NFs approach are not consistent with the results of the sc-CAFs-NFs approach, as well as with the existing data from the literature. Therefore, methods for obtaining CAFs *in vitro* require further research. It is also worth using this approach with caution when studying the features of the CAFs.

## 4. Discussion

Even though many studies over the years have suggested a prominent functional role for CAFs in tumor development (Kalluri, 2016; Zhuang et al., 2019; Sahai et al., 2020), there is still no clear definition of CAFs. As a consequence, the methods for their detection are not universal and not always precise enough (Ping et al., 2021). Inability of accurately isolating pure CAF population could be one of the reasons for existence of several studies with contradictory information about CAFs role in tumor development. Despite the fact that each of the CAF markers that exist in the literature has been repeatedly criticized, studies of the specificity of these markers based on a comprehensive analysis of gene expression in all tumor cells and their microenvironment have not yet been conducted. For this reason, in this study, we proposed a systematic approach for identification more specific CAF markers.

We have integrated many RNA sequencing data of CAFs and tumor microenvironment cells isolated from patients with different types of tumors obtained from various studies: 10 scRNA-seq datasets of different tumor tissues, mRNA-seq datasets of 934 human cancer cell lines from the Cancer Cell Line Encyclopedia, mRNA-seq datasets of 10000 patient tumor tissues from The Cancer Genome Atlas, 9 scRNA-seq of different normal tissues from Human Protein Atlas database. Through a comprehensive analysis of gene expression for 9 different types of tumors, we offer a list of 414 marker genes. Interestingly, 5 existing markers (*ACTA2, VIM, S100A4, POSTN, PDPN*) are not specific for any of the 9 tumor types we studied.

According to our analysis of the scRNA-seq data, we clearly detected 2 different clusters of cells expressed existing CAF markers (“Fibroblasts” and “Myofibroblasts/Mural cells”). It should be noted that the existence of several phenotypically and functionally different populations of CAFs within a single tumor has been repeatedly confirmed by many researchers both *in vivo* and *in vitro*, as well as in single-cell analysis of pre-sorted CAFs (Orimo et al., 2005; Li et al., 2017; Lambrechts et al., 2018; Zhang M. et al., 2020; Kieffer et al., 2020; Kim et al., 2020). In colorectal cancer, Li and colleagues identified two different populations of cancer-associated fibroblasts (CAF-A and CAF-B) based on RCA cell clustering (Li et al., 2017). They showed that CAF-A cells, in contrast to CAF-B, actively express genes *MMP2, DCN, COL1A2, CXCL12, FBLN1, LUM, FAP*. In turn, CAF-B cells are characterized by *ACTA2, TAGLN, PDGFA, MUSTN1, NOTCH3*, and *MYH11* genes. Bartoschek et al. identified four CAF populations in breast cancer: vascular CAFs, matrix CAFs, cycling CAFs and developmental CAFs (Bartoschek et al., 2018). However, many investigators studying the tumor microenvironment using scRNA-seq have detected multiple populations of CAFs due to the presence of other mesenchymal cells in the tumor with a similar expression profile to fibroblasts. For example, genes that are overexpressed in CAF-A and vascular CAFs have been proposed as distinctive markers of mural cells (Muhl et al., 2020). We believe that one of these cell clusters identified by our analysis as “fibroblast-like” consists of mural cells. It is confirmed by the fact that most genes specific to this cluster are actively expressed in mural cells according to HPA Single Cell Type Atlas. However, in accordance with the existence of several opinions concerning the annotation of fibroblasts and mural cells, we provided markers for both cell clusters we detected (Supplementary Tables S2–12).

Analysis of immunohistochemistry images from the Human Protein Atlas allowed us to identify those proteins among 414 our marker genes that are predominantly localized in the stroma of tumor tissue. As a result, we detected 10 universal proteins, whose expression are specific to fibroblasts in 9 tumor types (ASPN, PDGFRA, PDGFRB, MFAP5, CDH11, EMILIN1, COL12A1, COL6A3, NEXN, MFAP2). We provide information obtained from the analysis of scRNA-seq and immunohistochemistry images for all our proposed 414 markers for each type of tumor, so that the reader can independently choose the combination of markers that is most suitable for his study.

Moreover, to the best of our knowledge, we have for the first time compared several different approaches which allowed us to identify what changes fibroblasts undergo during the transformation from a normal to a tumor-associated state, regardless of the type of cancer. We found that scRNA-seq data from fresh tumor and normal tissues (sc-CAFs-NFs approach) are best suited to this task as it reveals the same gene changes between CAFs and NFs for several tumor types. Functional analysis of differentially expressed genes between CAFs and NFs revealed multiple pathways associated with extracellular matrix remodeling. These results support the current view that CAFs are a major component of the tumor stroma, which is involved in the organization of the tumor matrix (Sahai et al., 2020). It is known that CAFs actively produce matrix-crosslinking enzymes and ECM-degrading proteases, and thus, on the one hand, make a significant contribution to an increase in the stiffness of tumor tissue and, on the other hand, promote metastasis (Boire et al., 2005; Kalluri, 2016; Zeltz et al., 2020). In addition, differences in the expression levels of genes is associated with the various signaling pathways: PDGF, retinoic acid, interleukins, regulation of insulin-like growth factor, etc. Many studies have demonstrated that the activation of these signaling cascades in tumors promotes tumor growth and development. At the same time, we noticed the upregulation and downregulation of genes involved in several metabolic processes in CAFs. To the best of our knowledge, the critical role of CAFs as regulators of metabolic processes in cancer has been repeatedly confirmed by both *in vitro* and *in vivo* studies (Li et al., 2021). Increased production of glycosaminoglycans (including chondroitin sulfate) has been shown in some studies to be a feature of tumor fibroblasts and to promote proliferation and migration of tumor cells (Olsen et al., 1988; Fthenou et al., 2009; Wu et al., 2021). sc-CAFs-NFs analysis revealed a significant increase in CAFs expression of 3 exiting marker genes (*FAP*, *COL11A1* and *POSTN*) and 2 newly proposed markers (*COL6A3* and *MFAP2*) in all tumor types we studied. We consider that the MFAP2 protein is of the greatest interest for further study since it has membrane localization and can be used to isolate CAFs by FACS or magnetic particles. We also found that the expression profile of our proposed 414 marker genes can reliably distinguish between CAFs and NFs. Thus, changes in the expression of many of our proposed markers (including *COL6A3*, *MFAP2* and *MFAP5*) can indicate the transition of normal fibroblasts to a cancer-associated state (e.g., in *in vitro* experiments).

Surprisingly, the traditional approach (bulk-CAFs-NFs), which is based on identifying transcriptomic differences between fibroblasts isolated from tumor tissue and normal tissue located at some distance from the tumor, has been shown to be invalid. We observed no consistency in gene expression changes and no similarity in the biological processes behind the transcriptomic differences between CAFs and NFs in different cancer types. Furthermore, analysis of bulk RNA-seq data demonstrated that none of the currently used CAF markers (*ACTA2, FAP, POSTN, ASPN, MFAP5, PDPN, ITGA11, PDGFRA, PDGFRB, S100A4, VIM, COL11A1*) could specifically distinguish CAFs from NFs. Most probably, the results of the bulk-CAFs-NFs approach depend significantly on the methods used to isolate fibroblasts from the tissue, as well as the marker proteins used for isolation. As mentioned above, most of the controversies in CAFs studies have been attributed specifically to the use of different CAF populations. It is worth noting that the methodology of isolating fibroblasts from the tumor differed in each of the studies whose bulk RNA-seq data we used for analysis. Also, most of these studies did not indicate the distance from which the tumor normal fibroblasts were isolated. There is a lack of research into differences in the phenotype of fibroblasts close to the tumor to the ones that would be isolated from healthy donors. Another disadvantage of the bulk-CAFs-NFs approach is the need for *in vitro* additional cultivation to generate primary cell culture. In general, cells are grown for 2–10 passages before gene expression analysis. Although, through use of normal fibroblasts, it has been shown that cultivation significantly alters their morphology (Wang et al., 2015). Moreover, prolonged cultivation of normal fibroblasts can cause the gene expression changes (Neumann et al., 2010; Agorku et al., 2019; Machaliński et al., 2020). Our results also show that prolonged cultivation of normal skin fibroblasts significantly changes the expression levels of three fibroblast specific genes: *ASPN*, *PDGFRB* and *EMILIN1*. In addition, maintenance of a particular fibroblast phenotype is highly dependent on cultivation conditions. For example, culturing normal fibroblasts on high-stiffness polyacrylamide gels or increasing the concentration of fetal calf serum in the medium promotes a significant increase in ACTA2 expression and subsequent differentiation into myofibroblasts (Huang et al., 2012; Asano et al., 2017; Baranyi et al., 2019). In addition, it has not yet been shown that CAFs do not change their expression profile outside the tumor and its microenvironment (Sahai et al., 2020). Despite the common belief that a cancer-associated fibroblast phenotype remains stable *in vitro*, the opposite has already been demonstrated in some studies (Fuyuhiro et al., 2011; Wang et al., 2015). Thus, it is very difficult to understand whether the observed differences in gene expression obtained by bulk-CAFs-NFs approach are inferred from different fibroblast phenotypes or from the methods of cell isolation and cultivation conditions.

By analyzing data from the cult-CAFs-NFs approach we found that co-cultivation of normal fibroblasts with different tumor cell lines for 5-6 days significantly increased the expression of interleukins and chemokines in fibroblasts but did not alter the expression level of genes related to extracellular matrix remodeling. Moreover, the expression of *ACTA2*, *ASPN* and *COL11A1* genes are reduced in fibroblasts after cultivation with tumor cells. Although it has been repeatedly shown that the level of expression of these genes are much higher in tumor fibroblasts compared to fibroblasts from normal tissues *in vivo* (Bai et al., 2015; Jia et al., 2016; Sasaki et al., 2021). It is worth emphasizing that the challenges associated with *in vitro* cultivation discussed in the context of the bulk-CAFs-NFs approach are also relevant to the cult-CAFs-NFs approach. In this regard, due to the lack of reliable markers for CAFs as well as recognized distinguishing characteristics of NFs and CAFs, it is impossible to say at what point of co-cultivation with tumor cells, fibroblasts acquire the cancer-associated phenotype. In addition, there is currently no reliable information in the literature whether tumor cells alone are sufficient to cause fibroblasts to switch to a cancer-associated state. In the tumor microenvironment, fibroblasts are exposed to a variety of factors, including cell-cell interactions, hypoxic stress, nutritional deficiency etc. (Li et al., 2021; Ping et al., 2021). For example, hypoxic conditions, which are typical for the tumor microenvironment, have been found to activate the expression of *ACTA2* and some collagens in dermal fibroblasts (Zhao et al., 2017). Therefore, we could not claim that the transcriptional profiles of tumor fibroblasts and fibroblasts obtained by cultivation with tumor cells would be identical. We found that stimulation of normal fibroblasts with TNFα, which is actively expressed by tumor cells, leads to similar changes in the fibroblast transcriptome that are observed during co-cultivation with cancer cells. Nevertheless, the transcriptional profiles of CAFs and fibroblasts obtained by co-cultivation with tumor cells are not the same and results obtained using this method should be interpreted with caution.

To summarize, we have established a new method to identify CAF markers. The genes identified in this study are mostly different regarding currently used conventional markers. These genes can be used as novel markers to identify CAF population. Also, the comparison of several approaches to the analysis of the transcriptome of CAFs and NFs allowed us to explain the conflicting results of many studies devoted to the study of CAFs.

## Data Availability

The datasets presented in this study can be found in online repositories. The names of the repository/repositories and accession number(s) can be found in the article.
