# Supplementary material for: Deeper insights into transcriptional features of cancer-associated fibroblasts: An integrated meta-analysis of single-cell and bulk RNA-sequencing data
Source: Front Cell Dev Biol. 2022 Oct 3;10:825014. doi: 10.3389/fcell.2022.825014 (PMC9574913; doi:10.3389/fcell.2022.825014)
Supplement: Supplementary file 3 [file DataSheet1.pdf]

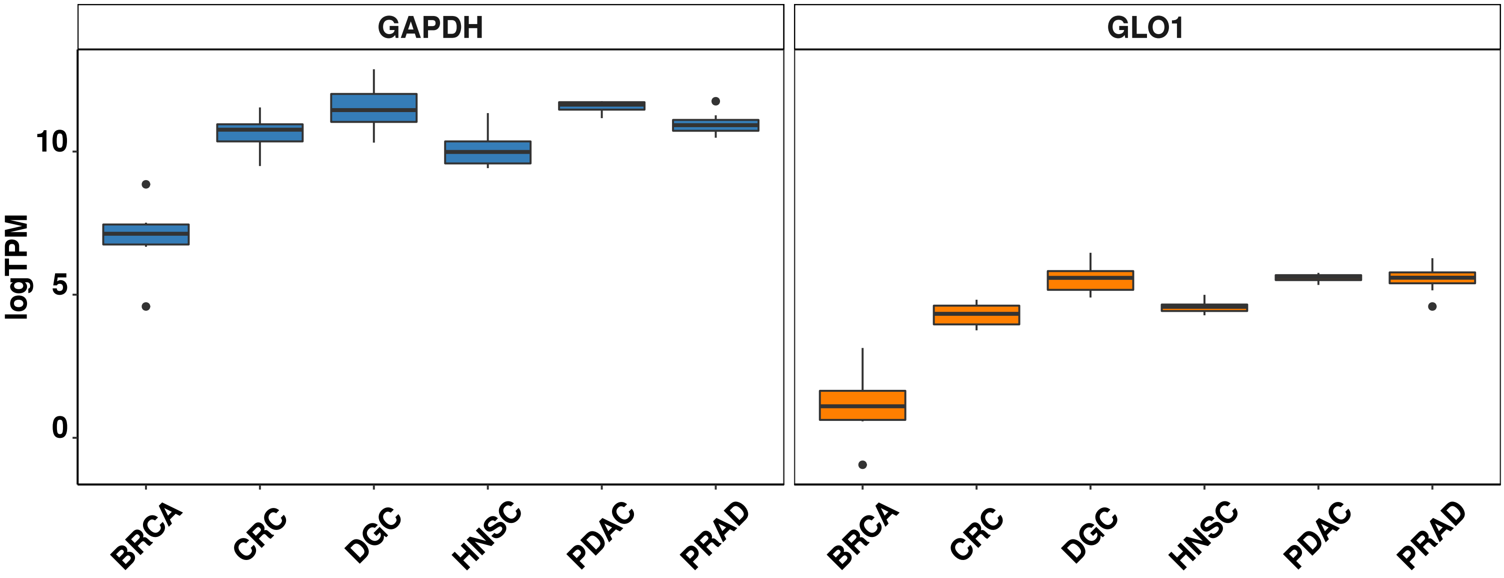

Supplementary Figure S1.
Evaluation of the commonly used reference gene GAPDH and candidate reference gene GLO1 based on bulk RNA-seq analysis of CAFs and NFs. A boxplot showing the log2(TPM) values of the GAPDH and GLO1 genes in different types of cancer. Box plots show quartiles, minimum, and maximum. Types of tumor: HNSC - head and neck squamous cell carcinoma, DGC - diffuse-type gastric cancer, BRCA - breast cancer, CRC - colorectal cancer, PRAD - prostate cancer, PDAC - pancreatic ductal adenocarcinoma.

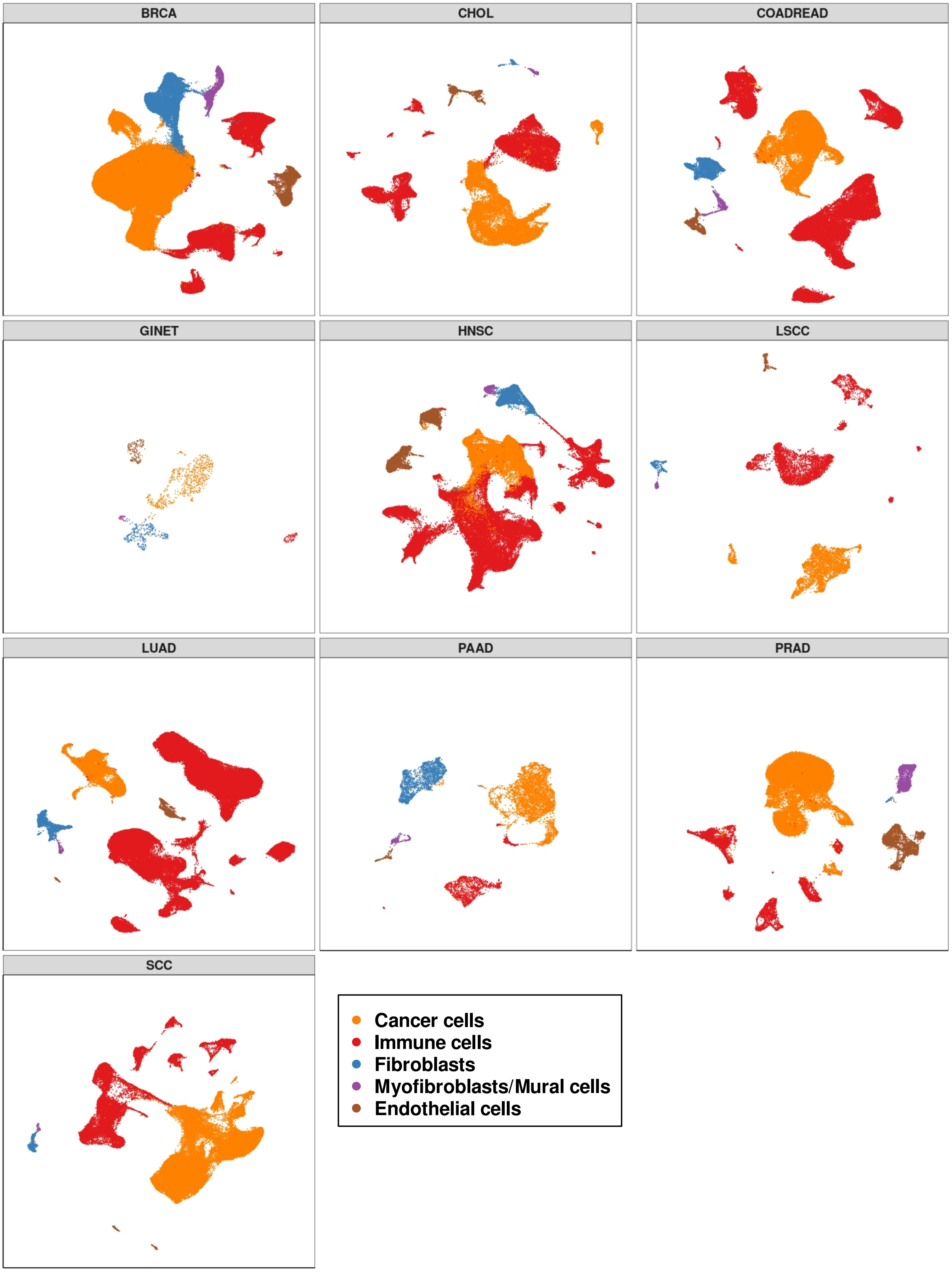

Supplementary Figure S2

Supplementary Figure S2 (previous page).

Cell clustering of different tumor samples and identification of cell types. UMAP plots of different tumor samples single cells demonstrate 5 distinct clusters of cells identified and color-coded. Orange = cancer cells, red = immune cells, blue = fibroblasts, purple = myofibroblasts/mural cells, brown = endothelial cells.

Types of tumor: BRCA - breast cancer, HNSC - head and neck squamous cell carcinoma, COADREAD - colorectal adenocarcinoma, LUAD - lung adenocarcinoma, PRAD - prostate adenocarcinoma, SCC - squamous cell carcinoma, CHOL - intrahepatic cholangiocarcinoma, LSCC - laryngeal squamous cell carcinoma, PAAD- pancreatic adenocarcinoma, GINET- gastrointestinal neuroendocrine cancer.

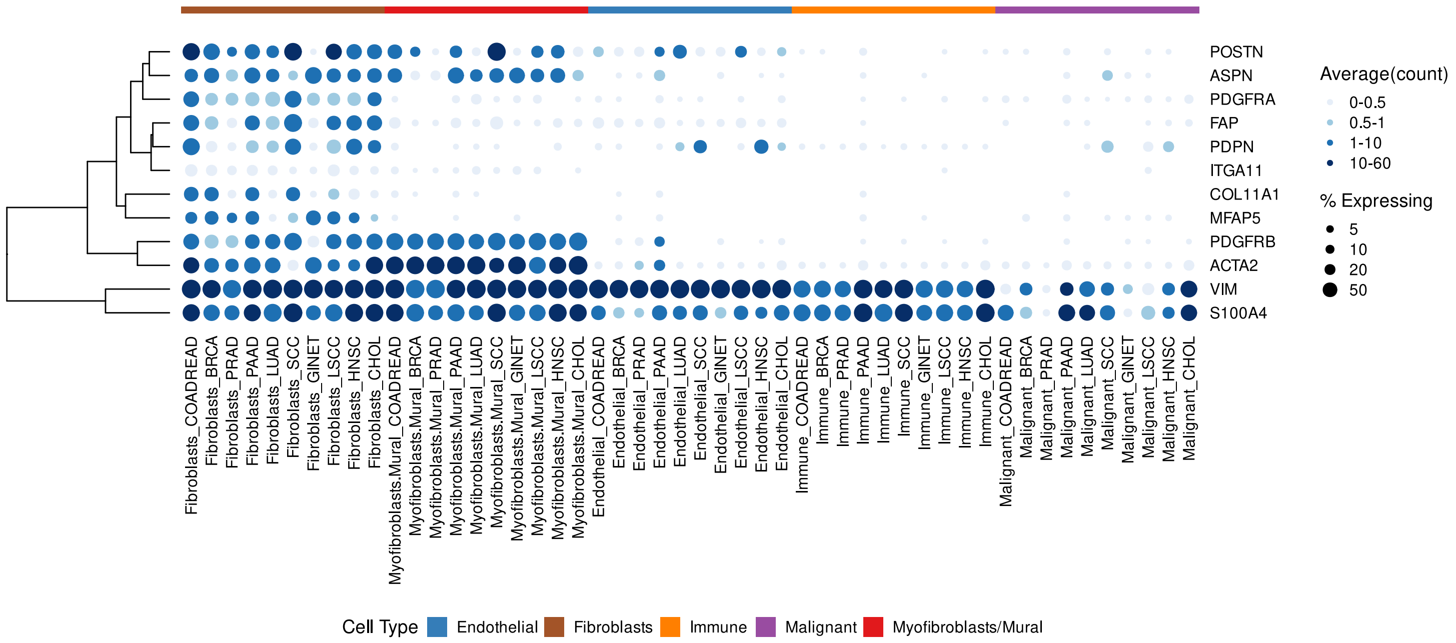

Supplementary Figure S3.

Visualization of common CAF marker gene expression across cell type and tumor type. Darker color indicates higher average gene expression from the cells in which the gene was detected, and larger dot diameter indicates that the gene was detected in greater proportion of cells from the cluster.

Types of tumor: BRCA - breast cancer, HNSC - head and neck squamous cell carcinoma, COADREAD - colorectal adenocarcinoma, LUAD - lung adenocarcinoma, PRAD - prostate adenocarcinoma, SCC - squamous cell carcinoma, CHOL - intrahepatic cholangiocarcinoma, LSCC - laryngeal squamous cell carcinoma, PAAD- pancreatic adenocarcinoma, GINET- gastrointestinal neuroendocrine cancer.

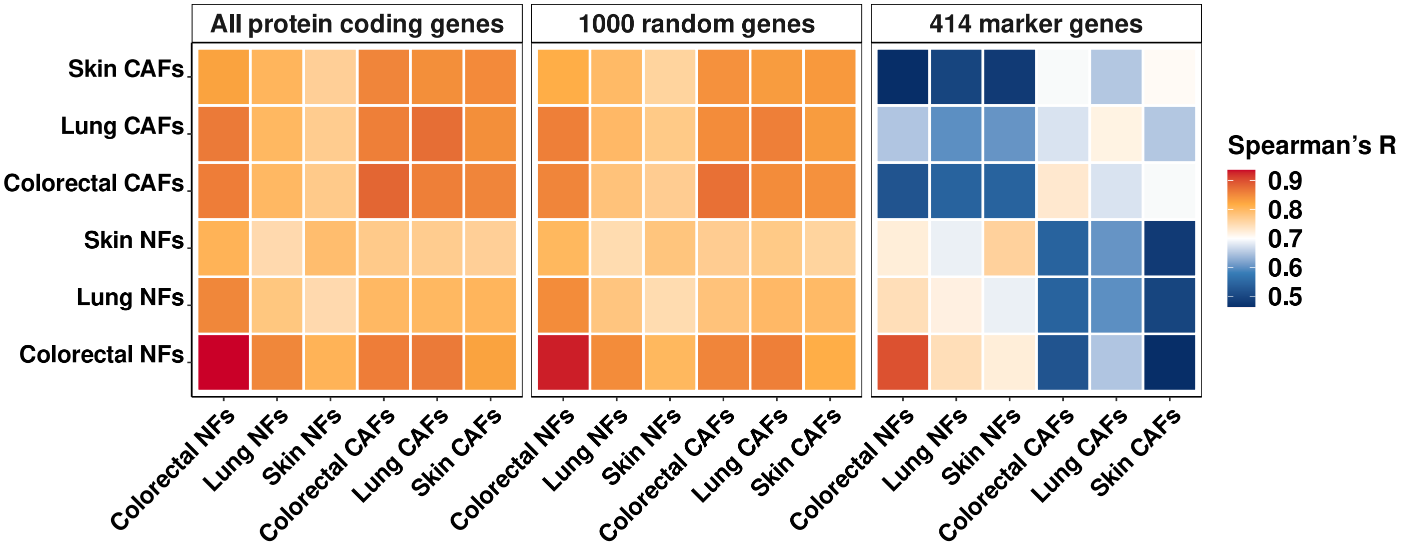

Supplementary Figure S4.
The Spearman's correlation analysis of gene expression profiles of NF and CAF samples (all protein coding genes, 414 CAF marker genes and 1000 random genes) (sc-CAFs-NFs approach). Spearman's R values are the medians of all pairwise Spearman's correlation coefficient values between samples.

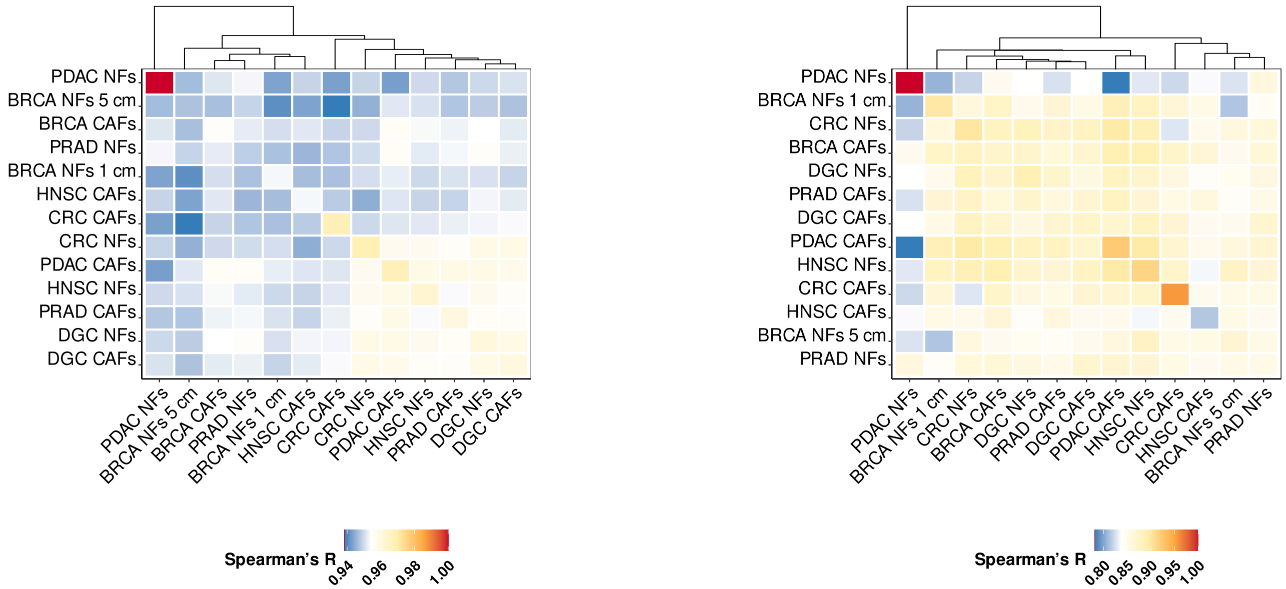

Supplementary Figure S5.
The Spearman's correlation analysis of gene expression profiles of NF and CAF samples (bulk-CAFs-NFs approach). A - Correlation based on the level of expression of all protein-coding genes. B - Correlation based on the level of expression of 414 CAF marker genes. Spearman's R values are the medians of all pairwise Spearman's correlation coefficient values between samples.

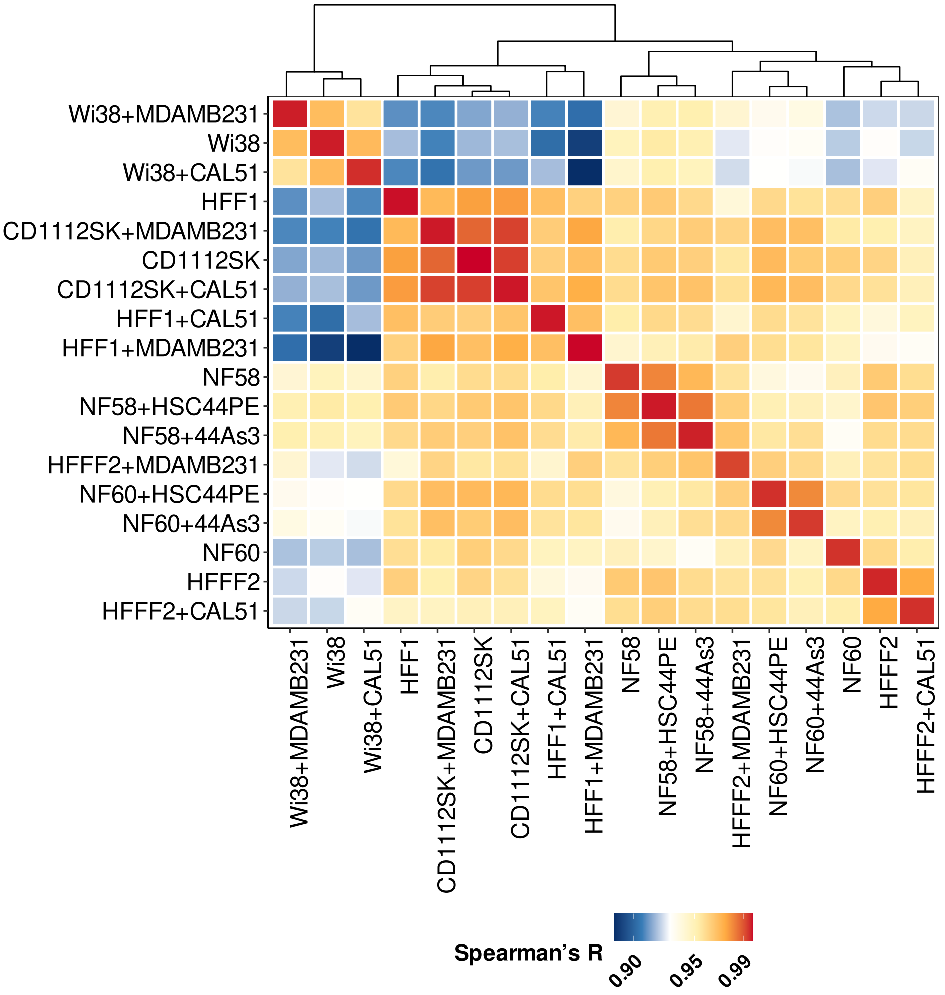

Supplementary Figure S6.
The Spearman's correlation analysis of gene expression profiles of NF and CAF samples based on the level of expression of all protein-coding genes (cult-CAFs-NFs approach). Spearman's R values are the medians of all pairwise Spearman's correlation coefficient values between samples.
